# Peri-exposure protection against Nipah virus disease using a single-dose recombinant vesicular stomatitis virus-based vaccine

**DOI:** 10.1038/npjvaccines.2016.2

**Published:** 2016-07-28

**Authors:** Blair L DeBuysscher, Dana Scott, Tina Thomas, Heinz Feldmann, Joseph Prescott

**Affiliations:** 1 Laboratory of Virology, Division of Intramural Research, National Institute of Allergy and Infectious Diseases, National Institutes of Health, Rocky Mountain Laboratories, Hamilton, MT, USA; 2 Division of Biological Sciences, University of Montana, Missoula, MT, USA; 3 Rocky Mountain Veterinary Branch, Division of Intramural Research, National Institute of Allergy and Infectious Diseases, National Institutes of Health, Rocky Mountain Laboratories, Hamilton, MT, USA

## Abstract

Nipah virus is a zoonotic paramyxovirus that causes severe disease in humans and animals. Due to almost yearly outbreaks in Bangladesh, and a large outbreak in Malaysia that lead to the shutdown of swine export, Nipah virus is both a threat to public health and the economy. Infection is associated with respiratory distress, encephalitis and human-to-human transmission, resulting in high case fatality rates during outbreaks. This study aims to address the amount of time needed until protection from a recombinant vesicular stomatitis virus-based vaccine candidate expressing the Nipah virus glycoprotein (G), which we have previously shown to protect hamsters and non-human primates when administered 28 days before challenge. We found that a single-dose vaccination, when administered 1 day before challenge, reduced viral load, limited pathology and fully protected hamsters from Nipah virus infection. The vaccine was even partially protective when administered at early time points following challenge with Nipah virus. These data indicate that a single administration of this vaccine to high-risk individuals, such as family members and health-care workers of infected patients, could be protective and useful for reducing human-to-human transmission and curbing an outbreak.

## Introduction

Nipah virus is a negative-stranded RNA virus in the *Paramyxoviridae* family and is a zoonotic pathogen with a broad species tropism.^1^ The virus was discovered during an outbreak of respiratory and neurologic disease in swine and humans in Malaysia in the late 1990s.^2^ Epidemiological investigations from the outbreak lead to the identification of fruit bats of the *Pteropididae* family as the reservoir for this virus.^3,4^ The outbreak was contained by stopping the export of pigs in and around Malaysia, culling over a millions pigs, and the quarantine of swine farms and workers.^5^ These steps helped end the outbreak, but also resulted in economic losses to the Malaysian pig industry.

After the initial outbreak, Nipah virus did not re-emerge until 2001, where it was responsible for an outbreak in Bangladesh.^6^ Since this first Bangladeshi outbreak, there have been almost yearly re-emergences of Nipah virus in Bangladesh or India. In Bangladesh, the outbreak was confined to the human population and lacked the swine intermediate. Instead of transmission from bats to swine and subsequently humans, transmission of Nipah virus in Bangladesh is not thought to involve an intermediate host. Here, transmission to humans has been hypothesised to be from contact with bat excrement, consumption of bats or collection and consumption of date palm sap that is contaminated with Nipah virus.^7–9^ Disease resulting from the outbreaks of Nipah virus in Bangladesh is similar to that of the Malaysian outbreak, but is associated with increased human-to-human transmission, a more pronounced respiratory component, and a higher case fatality rate; up to 100% in individual small outbreaks, with an average of 70%.^10,11^ Both outbreak regions involved cases of relapsing encephalitis.^12^

Nipah virus disease is characterised by virus-induced lesions, syncytia and viral antigen in humans, causing cytopathology and immunopathology in effected organs.^13,14^ Viral tropism is dictated by the tissue distribution of the entry receptor (ephrin B2/B3), which is expressed at high levels in endothelial cells, neurons and epithelial cells.^1,15,16^ Nipah virus antigen and RNA are most often found in the lungs, brain, spleen and replication leads to pathologies, including acute pneumonia and encephalitis.^1^ In the lungs, pathology is most often associated with the microvasculature, causing inflammation (vasculitis) and necrosis.^17^ Similar pathology is seen in the brain and is associated with neurons.^18,19^

There have been various attempts to develop vaccines against Nipah virus that have been tested in animal models. Approaches include multiple platforms focused on the glycoproteins (fusion (F) and glycoprotein (G)) as immunogens, and can be separated into two categories based on either administration of a single dose or a prime-boost approach.^20–33^ Our laboratory has shown that a single dose of live-attenuated, recombinant vesicular stomatitis virus (rVSVs) vectors expressing the Ebola virus glycoprotein and one of the Nipah virus glycoproteins (G or F; rVSV-EBOV-GP-NiV-G, rVSV-EBOV-GP-NIV-F) are fully protective in the hamster model when administered 28 days before challenge with 1,000 times the 50% lethal dose (LD_50_) of Nipah virus.^32^ We have also demonstrated complete protection by the rVSV-EBOV-GP-NiV-G vaccine against Nipah virus disease in the African green monkey model with a single dose of this vaccine.^34^

In the study herein, we aimed to define the time to protection after vaccination, and to determine whether this vaccine can provide post-exposure protection. A vaccine that provides rapid protection when administered around the time of exposure/infection (peri-exposure) will be suitable for emergency use in a Nipah outbreak situation, both for ring vaccination strategies, and to protect health-care workers entering outbreak regions on short-notice deployment. We show that our rVSV-EBOV-GP-NiV-G vaccine is fully protective in the hamster model when given as late as 1 day before challenge, and provides partial protection when administered up to a day after challenge. Protection correlated with reduced viral loads and virus-associated pathology in target tissues. These data show that our vaccine could be used to elicit a rapid and robust immune response that could protect high-risk individuals in case of outbreak emergencies.

## Results

### Vaccination shortly before challenge is protective

To test the window of protection of the rVSV-EBOV-GP-NiV-G vaccine, we vaccinated animals at time points within 1 week surrounding challenge with Nipah virus. Animals were challenged with 1,000 LD_50_ of Nipah virus—Malaysia intraperitoneally. Animals were monitored for signs of disease throughout the experiment and euthanized when respiratory distress or neurologic signs of disease were observed.

**Figure 1:**
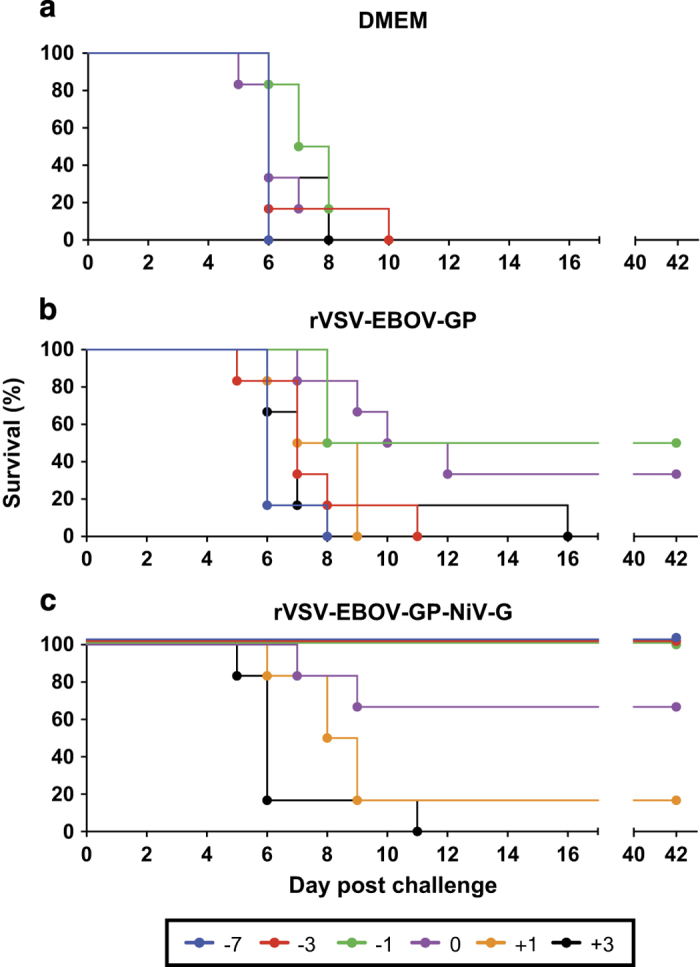
Survival of vaccinated hamsters following Nipah virus challenge. Groups of six hamsters were vaccinated intraperitoneally with 10^5^ plaque-forming units of the designated vaccine; (**a**) DMEM control, (**b**) rVSV-EBOV-GP and (**c**) rVSV-EBOV-GP-NiV-G. Individual vaccines were administered at the indicated time points relative to challenge. Hamsters were challenged with 1,000 LD_50_ of Nipah virus—Malaysia and monitored for disease for 42 days.

As expected, animals administered Dulbecco’s modified Eagle medium (DMEM) as a control succumbed to infection between days 6 and 10 after challenge (Figure 1). Interestingly, the vaccine vector control (rVSV-EBOV-GP) showed partial protection when administered close to the time of challenge, with 50% surviving when vaccinated 1 day before challenge and 33% survival when the backbone vector was given an hour after challenge (Figure 1). Other time points, either earlier or later with respect to challenge, showed that no protection was afforded by the vector control. Animals vaccinated with rVSV-EBOV-GP-NiV-G as little as 1 day before challenge were fully protected against Nipah virus disease (Figure 1). Animals vaccinated an hour after challenge (0 day) were partially protected with four out of six (67%) animals surviving challenge. Vaccination 1 day after challenge protected only one out of six animals (17%), whereas no animals from the +3-day group survived. Animals that were protected in an individual group did not show any signs of disease or distress throughout the duration of the experiment.

### Survivors generate antibodies against Nipah virus

**Figure 2:**
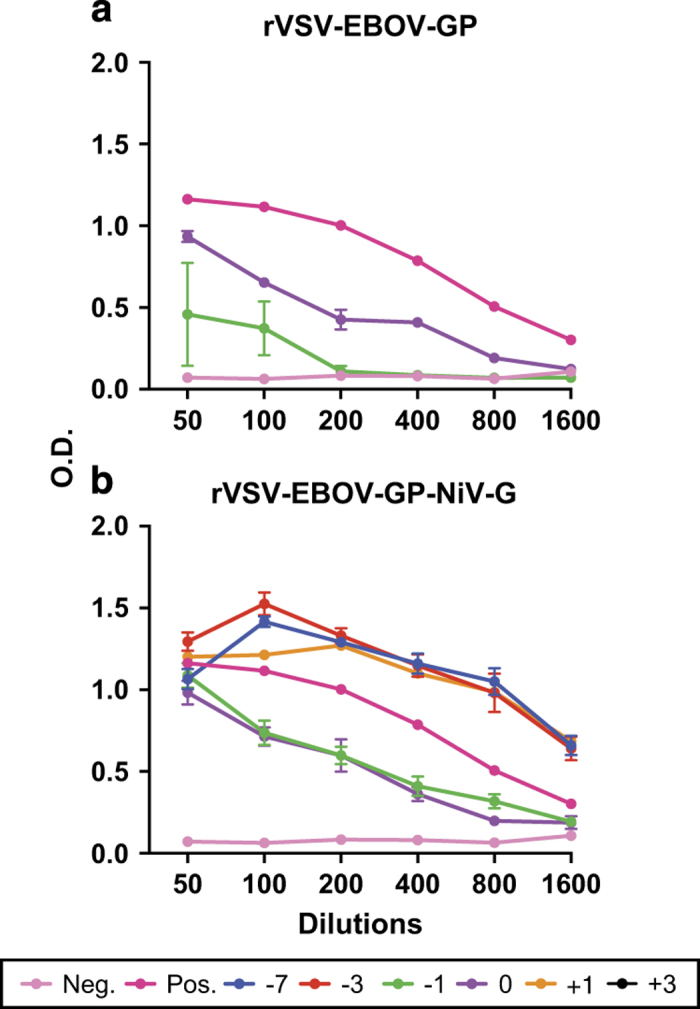
Antibody titres to Nipah virus in hamsters after challenge. Sera were collected from hamsters at 42 d.p.i. and a Nipah virus-specific enzyme-linked immunosorbant assay was performed. (**a**) Survivors vaccinated with rVSV-EBOV-GP vector control and (**b**) survivors vaccinated with rVSV-EBOV-GP-NiV-G. Colours represent the time of vaccination and data are shown as an average of all animals in a particular group that remained at 42 d.p.i. The negative control is non-infected hamster serum and the positive control serum was from a Nipah-infected hamster.

Blood was collected at the time of euthanasia (42 d.p.i.), for measuring antibody responses. All hamsters tested had measurable antibody titres to whole Nipah virus by enzyme-linked immunosorbent assay, showing that vaccination and/or challenge evoked an antibody response. The rVSV-EBOV-GP-NiV-G vaccinated survivors had slightly higher responses compared with vector controls (Figure 2).

### Decreased Nipah virus loads in tissues correlates with protection

**Figure 3:**
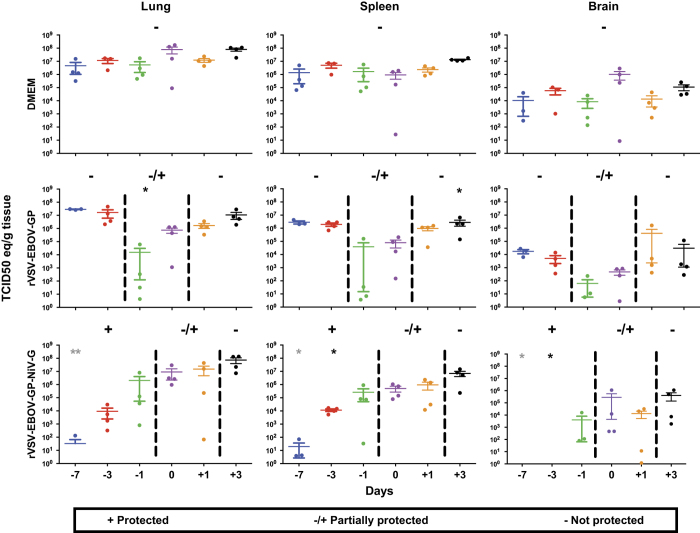
Decreased viral load in tissues is associated with survival. Tissues (lung—left column, spleen—middle column and brain—right column) from four animals per group were collected 5 days after challenge. Tissues were homogenised and total RNA was extracted. TCID_50_ equivalents were determined by quantitative reverse transcription PCR using an N gene-specific primer and probe set with a standard of known TCID_50_ equivalents. Individual animals from each vaccination group (DMEM—top row, rVSV-EBOV-GP—middle row and rVSV-EBOV-GP-NiV-G—bottom row) are represented by individual dots, bars represent the mean of each group and error bars indicate s.e.m. Groups of animals that were protected by vaccination in the survival experiment are designated by (+), partially protected by (±) and groups not protected by (−). One-way analysis of variance with Tukey’s post tests was performed on each vaccination time point and significance is represented by asterisks (*P⩽0.05 and **P⩽0.01). Black asterisks denote significance between the vaccinated groups and DMEM controls, and grey asterisks represent differences between the vaccinated groups and vector controls.

Tissues were collected from each of four animals in each group 5 days after challenge to determine the impact of vaccination on Nipah virus replication. RNA was extracted from the brains, lungs and spleens for use in a quantitative reverse transcription PCR assay targeting the Nipah virus N gene. Tissues from the DMEM animals had consistently high viral loads, with over 10^5^ tissue culture infectious dose-50% (TCID_50_) equivalents in the lungs and spleens, and over 10^3^ TCID_50_ in brain tissue (Figure 3, top row). Like the DMEM controls, animals in the rVSV-EBOV-GP group that did not survive infection for a specific time point had high viral loads in these tissues. A decrease in viral load was seen in the groups corresponding to partial protection, which include days −1 and 0 (Figure 3, middle row), due to the variation between animals this was not significant. RNA loads from the non-surviving rVSV-EBOV-GP-NiV-G vaccinated groups were consistent with non-survivors from the other groups. Animals from the groups that corresponded to partial protection (days 0 and −1) had intermediate levels of RNA compared with the protected and non-protected groups. There was a high variability in viral loads between animals at 5 d.p.i. in the groups that showed partial protection in the corresponding survival experiments. This suggests that the animals with lower viral loads likely are representative of the survivors in these groups. Similar to what was observed in our previous study with vaccination at 28 days before challenge,^32^ the fully protected groups vaccinated with rVSV-EBOV-GP-NiV-G had highly reduced viral loads, exhibiting a significant difference between both control and vector only vaccination at the corresponding time points as shown in Figure 3 (bottom row). Some animals within the completely protected groups had no detectible viral RNA.

### Protected animals have reduced pathology

**Figure 4:**
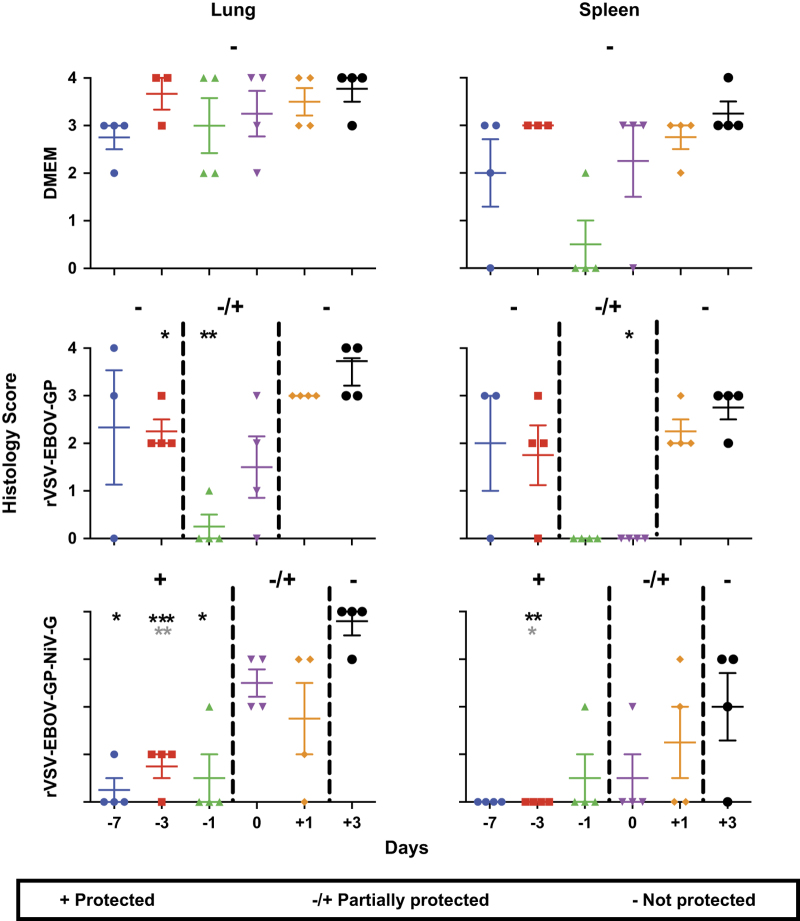
Decreased pathology in the spleen and lungs corresponds to increased survival. Pathology scores ranging from 0 to 4 were assigned to the lung and spleen sections from each group of four animals collected at 5 days after challenge. Results of clinical scoring of brain tissue are not shown because no changes were observed. Groups of animals that were protected by vaccination in the survival experiment are designated by (+), partially protected by (±) and groups not protected by (−). Bars represent the mean of each group and error bars indicate s.e.m. Kruskal–Wallis analysis with a Dunn’s post test was performed, and significance is represented by asterisks (*P⩽0.05, **P⩽0.01 and ***P⩽0.001). Black asterisks denote significance between vaccinated groups and DMEM controls, and grey asterisks represent differences between the vaccinated groups and vector controls. The scoring criteria were as follows: 0=no lesions; 1=focal to few multifocal mild lesions; 2=multiple multifocal mild-to-moderate lesions; 3=multifocal areas of moderate-to-severe lesions; and 4=extensive areas of lesions and necrosis.

**Figure 5:**
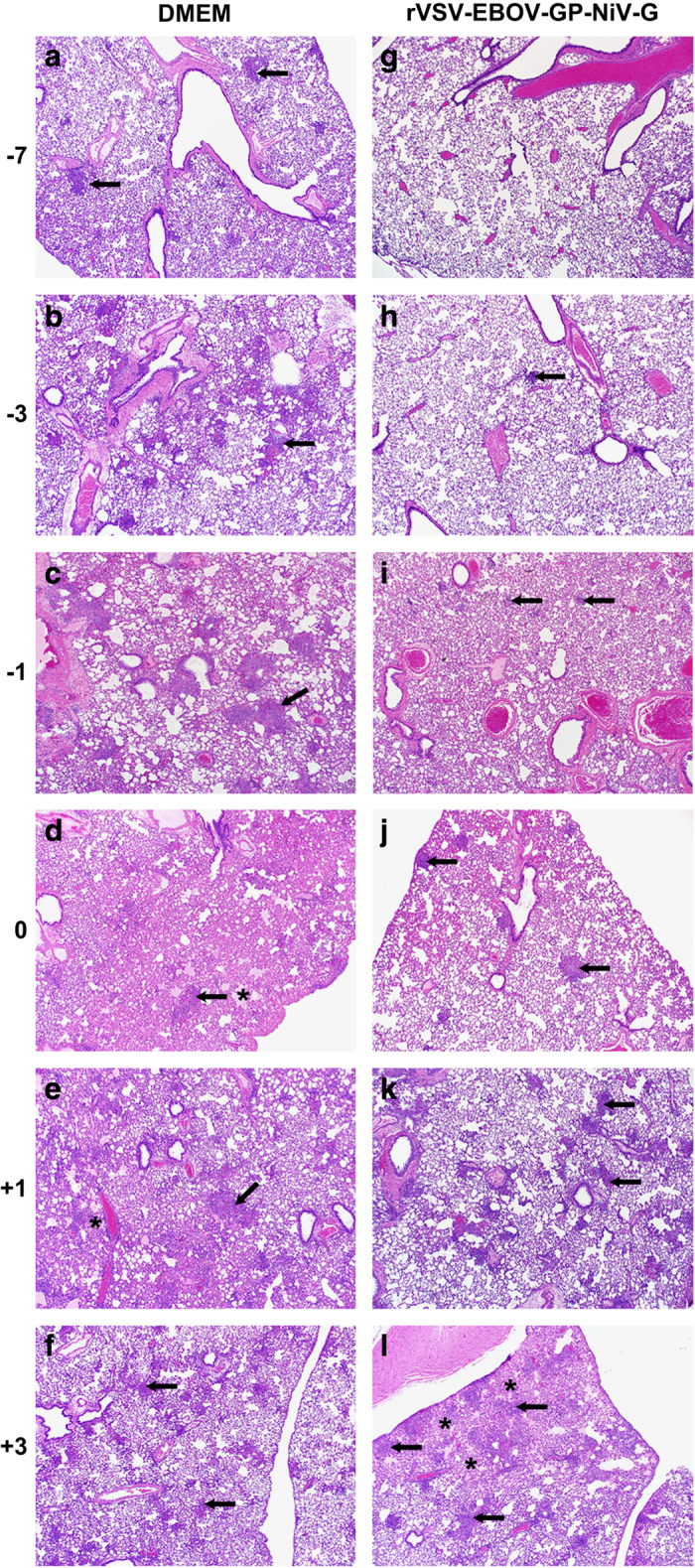
Protective vaccination is associated with reduced Nipah virus pathogenesis. Lung sections from four hamsters per group were collected 5 days after challenge and stained with H&E for histology. (**a**–**f**) Taken from representative, unprotected DMEM control animals and show moderate-to-marked interstitial pneumonia with necrosis and inflammation (arrows), and alveolar oedema and fibrin (asterisk). (**g**–**l**) Derived from rVSV-EBOV-GP-NiV-G animals. (**g**) An example of normal tissue from a protected animal. (**h**, **i**) Minimal interstitial pneumonia with rare foci of alveolar interstitial inflammation (arrow). (**j**) Mild interstitial pneumonia with larger more frequent areas of inflammation (arrows). (**k**, **l**) Animals with moderate-to-marked interstitial pneumonia with coalescing inflammation in **k**, and necrosis, inflammation and alveolar oedema, and fibrin (asterisk) in **l**.

To assess the pathogenic effects of Nipah virus on the various groups, samples of the brain, lung and spleen were collected on day 5 after challenge for histopathologic examination. Tissues were scored from 0 to 4 according to the parameters in the Materials and Methods section, with 0 being a lack of pathology and 4 representing severe pathology. The average scores for the lung and spleen are represented in Figure 4; however, the brain was excluded because no pathologic changes were observed. As expected, DMEM control animals had high scores in lung tissue samples at all time points. Moderate-to-marked multifocal to coalescing interstitial pneumonia was prominent (Figure 5). Pneumonia was characterised by thickening of the alveolar interstitium by oedema, fibrin, as well as inflammatory cell infiltrate and necrosis. Alveolar spaces in the affected areas were filled with haemorrhage, oedema, fibrin, neutrophils and macrophages. Multifocal vasculitis of small and medium calibre arteries was also present.

Animals in the protected groups had low pathological scores, or no score, showing clear airways and only rare lesions (Figures 4 and 5). Specifically, animals vaccinated 7 days before challenge demonstrated no lesions. As vaccination approached the time of challenge (days −3 and −1), hamsters developed minimal interstitial pneumonia with few foci of alveolar interstitium expansion and clear alveolar spaces with no evidence of vasculitis. Animals from the partially protected group vaccinated shortly after challenge (day 0) had similar lesions to those vaccinated on days −3 and −1, with the affected area being larger and more frequent. Hamsters vaccinated 1 day after challenge (+1) showed mixed levels of pathology, with half of the group developing lesions similar to those seen in the protected groups and the other half developed intestinal pneumonia that was indistinguishable from unprotected controls. The unprotected group vaccinated 3 days after challenge (+3) had pathology that mirrored animals of the unprotected control group. In addition to lung pathology, animals in the control group and groups vaccinated after challenge, developed mild-to-moderate splenic vasculitis with perivascular fibrin and lymphoid necrosis of the white pulp.

## Discussion

Nipah virus infects humans in the Indian sub-continent, causing severe disease and subsequent death in ~70% of cases.^35^ Disease is characterised by respiratory distress and/or encephalitis, and currently there is no approved human vaccine against Nipah virus; however, a subunit vaccine has been approved for the use in horses against the closely related Hendra virus in Australia.^26^ Previous studies have focused on protection against disease using neutralising antibodies, subunit vaccines based on glycoproteins, virus-like particles, DNA vaccination and various vectored vaccine platforms.^20–25,27–33^ Most of these vaccine approaches require boost immunisations for protection, with repeated administration over many weeks or months. Thus far, there has been little focus on examining or reducing the time to protection afforded by these vaccines, thus promoting a vaccine’s usefulness as an emergency countermeasure during an outbreak.

We aimed to characterise the rVSV-EBOV-GP-NiV-G vaccine, which has previously been shown to elicit a protective response in African green monkeys and hamsters when administered a month before challenge.^32,34^ In the study herein, we sought to determine the minimum time between vaccination and challenge that is required to elicit a protective response, and to determine whether this vaccine can afford protection when given after challenge. By investigating the window of time needed for protection, we will be able to explore the possibility of vaccination both during outbreaks, as well as for laboratory exposures. We used an attenuated replicating virus vector, which has the ability to stimulate innate and adaptive immune responses making it a possible candidate for peri-exposure vaccination.^36–39^ The rVSV-EBOV-GP vector, which is the backbone vector for our Nipah vaccine used herein, has previously been shown to protect against both Ebola virus and Andes virus when administered around the time of virus challenge in hamsters.^36,40^ In non-human primates, the rVSV vector encoding the GP of Marburg virus was almost completely protective when administered 1 day after challenge, and partially protective when administered 2 days after challenge.^41^ This is likely due to the cell types targeted by the glycoprotein of filoviruses, such as macrophages, monocytes and dendritic cells, as well as the ability of the replication-competent recombinant VSV to elicit a strong and rapid immune response.^42,43^

In our study, we found that the rVSV-EBOV-GP vector alone, when administered either the day before or an hour after challenge, elicited partial protection against Nipah virus and inhibited virus replication. A likely explanation for this observation is that the recombinant replicating vector acts by stimulating the host’s innate immune response to a level that tempers replication and causes a delay in the pathogenic process, giving time for the development of a virus-specific adaptive immune response. Our laboratory has previously shown that this vector encoding the GP of EBOV stimulates high levels of transcription of the innate immune response genes Mx-2 and STAT-1 in the lungs and spleens of hamsters, with maximal transcription levels at 1 day after vaccination and reduced levels at 3 days after vaccination.^36^ We have also shown that the rVSV-EBOV in a non-human primate model led to elevated interferon (IFN) α, IFNγ, interleukin (IL)-15, IL-6 and transforming growth factor-α, shortly after administration of the vaccine.^37^ The increase of these cytokines is suggestive of macrophage and natural killer cell activation, thus eliciting an early innate anti-viral state, tempering viral replication. Stimulation of the innate immune response in hamsters, using synthetic double-stranded RNA (poly I:C), has been shown to be protective against Nipah virus disease when given post inoculation and for 10 days thereafter, suggesting that prolonged simulation of innate immunity alone is sufficient for protection.^44^ This effect, combined with the incorporation of the Nipah virus G into the vaccine vector, led to full protection when the vaccine was administered just before challenge, and partial protection was observed as late as 1 day after challenge. The mechanism of added protection in the rVSV-EBOV-GP-NiV-G vaccine groups over vector alone probably is its potential to induce early Nipah-specific antibody responses.

Current outbreaks of Nipah virus involve human-to-human transmission, mostly involving interfamily interactions. This vaccine, which elicited full protection as early as 1 day before exposure in hamsters, would be a candidate for ring vaccinations during outbreaks, both for health-care workers and high-risk individuals. As Nipah virus has a longer incubation period in humans (average of ~2 weeks) compared with hamsters (~6–8 days at the dose used), there would possibly be a larger time window after infection for the protective effects of this vaccine in humans, due to this delay in pathogenesis.^11,18,45,46^ Similarly, the African green monkey model provides a means to better define the peri-exposure effectiveness of this vaccine, as animals typically develop severe disease that require euthanasia between days 9 and 12, depending on the dose of Nipah virus used, and this hypothesis could be tested by performing similar experiments as done herein using non-human primates.^34,47^ The African green monkey would also help to satisfy guidelines put forth by the Food and Drug Administration for testing vaccines.^48^

Overall, we demonstrate that our attenuated, recombinant replication-competent vaccine is fast-acting producing a sufficient protective response that leads to survival in hamsters, even when administered as late as the day before challenge, and has some efficacy when given after challenge. This vaccine is a potential candidate for an effective human Nipah virus vaccine. The use of rVSV-vectored vaccines for humans is supported by current clinical trials of rVSV-EBOV-GP, which is the backbone of the Nipah vaccine used here, in West Africa to combat the current Ebola virus outbreak.^49,50^

## Materials and Methods

### Biosafety and ethics

Animal experiments were performed under the guidelines of the Association for Assessment and Accreditation of Laboratory Animal Care, International (AAALAC) in an AAALAC-approved facility by certified staff. Work with Nipah virus was done under standard operating procedures approved by the Institutional Biosafety Committee in the BSL-4 facility at the Rocky Mountain Laboratories, National Institutes of Allergy and Infection Diseases, National Institutes of Health.

### Cells and viruses

Vero C1008 cells (European Collection of Cell Cultures, Salisbury, UK) were used to propagate Nipah virus.^46^ Nipah virus—Malaysia was kindly provided by the Special Pathogens Branch of the Centers for Disease Control and Prevention, Atlanta. rVSV-EBOV-GP-NiV-G was generated as described previously.^32^

### Immunisation, challenge and monitoring in Syrian hamsters

Groups of 10 female 5–6-week-old Syrian hamsters (Harlan Laboratories, Indianapolis, IN, USA) were administered 10^5^ plaque-forming units of rVSV-EBOV-GP-NiV-G, sterile medium as a control (DMEM) or the backbone vector (rVSV-EBOV-GP) intraperitoneally in a volume of 500 μl. Groups were vaccinated on days −7, −3 or −1 before challenge or day 0 (+1 h), +1 or +3 after challenge. Animals were challenged intraperitoneally with 1,000 LD_50_ (6.8×10^4^ TCID_50_) of Nipah virus—Malaysia on day 0. After challenge, the hamsters were monitored daily for signs of disease using a scoring system approved by the Institutional Animal Care and Use Committee. Four pre-determined animals per group were killed 5 days after challenge with Nipah virus to measure viral loads and to assess histopathologic changes. Tissues (brain, lung and spleen) were collected in RLT lysis buffer (Qiagen, Valencia, CA, USA) for RNA extraction or 10% formalin for histopathology. The remaining six animals in each group were monitored for 42 days after challenge for clinical signs of disease and survival.

### Enzyme-linked immunosorbant assay

Serum was collected at the time of euthanasia and assessed for the presence of anti-Nipah virus antibodies by enzyme-linked immunosorbant assay as described previously.^51^

### Nipah virus quantitation

Viral loads in tissues were measured by quantitative real-time reverse transcription PCR targeting the nucleoprotein (N) gene of Nipah virus.^46^ RNA values were determined using defined RNA dilutions in triplicate to create a standard curve, which allowed for the extrapolation of TCID_50_ equivalents.

### Histopathology

Tissues were fixed in 10% formalin for 7 days with one volume change before being transferred out of the BSL-4, following standard operating procedure approved by the Institutional Biosafety Committee. Tissues were then placed in cassettes and processed with a VIP-5 Tissue Tek (Sakura Finetek, Torrance, CA, USA), on a 12 h automated schedule, using a graded series of ethanol, xylene and ParaPlast Extra. Five micrometre sections of embedded tissue were dried overnight at 42 °C, then staining with haematoxylin and eosin (H&E). H&E slides were scored blindly according to the following criteria: 0=no lesions; 1=focal to few multifocal mild lesions; 2=multiple multifocal mild-to-moderate lesions; 3=multifocal areas of moderate-to-severe lesions; and 4=extensive areas of lesions and necrosis.

### Statistics

For the determination of significance for the pathology scores, one-way analysis of variances with Tukey’s post tests were performed on each vaccination time and Kruskal–Wallis with a Dunn’s post test was performed for the viral load data.
